# Carotid vulnerable plaques are associated with circulating leukocytes in acute ischemic stroke patients: an clinical study based on contrast-enhanced ultrasound

**DOI:** 10.1038/s41598-018-27260-0

**Published:** 2018-06-11

**Authors:** Zhaojun Li, Yun Bai, Wanbin Li, Feng Gao, Yi Kuang, Lianfang Du, Xianghong Luo

**Affiliations:** 1 Department of Ultrasound, Shanghai General Hospital, Shanghai Jiaotong University School of Medicine, Shanghai, China; 2 Department of Echocardiography, Shanghai General Hospital, Shanghai Jiaotong University School of Medicine, Shanghai, China

## Abstract

Inflammatory activity plays a central role in the development of carotid rupture-vulnerable atherosclerotic plaques, which is one of the major contributors to acute ischemic stroke. Our objective was to characterize carotid intraplaque neovascularizations (INP) using contrast-enhanced ultrasound (CEUS) and evaluate plaque burden through exploring the relationship between INP and cell count of peripheral leukocytes. Sixty-two patients with large artery atherosclerosis (LAA) were enrolled in this study. CEUS was performed to characterize the carotid artery plaques. The correlations between the CEUS imaging features of carotid plaques and leukocyte counts were investigated. The results showed that the characteristic parameters derived from CEUS, including peak of time-intensity curve (TIC-P), mean of time-intensity curve (TIC-M), peak (FC-P), sharpness (FC-S) and area under the curve (FC-AUC) compared with the control group, were all increased in the stroke group. TIC-P, TIC-M and FC-P were negatively related to lymphocytes, respectively. FC-S and FC-AUC were positively correlated with neutrophils, respectively. Our study indicated carotid INP was closely related to the peripheral leukocytes count. CEUS may serve as a useful tool to predict vulnerability of plaque.

## Introduction

Stroke is one of the leading causes of mortalities worldwide with 15 million people experiencing a new or recurrent stroke every year, resulting in 5 million deaths and an additional 5 million patients who are permanently disabled. Acute ischemic stroke (AIS) accounts for about 80 percent of all stroke^1^. Around 20% of ischemic strokes appear to originate from carotid plaques^2^. According to the Trial of Org 10172 in Acute Stroke Treatment (TOAST) Subtype Classification System, AIS is more likely to be associated with the rupture of carotid plaque^3^. An important characteristic of vulnerable plaques is increased macrophage content. A high rate of oxygen consumption by plaque macrophages causes hypoxia within the plaque, which induces a continual release of growth factors that stimulate the neovascularization processes^4,5^. The neovascularization in the plaque eventually contribute to the instability of the plaque, leading to intraplaque hemorrhage, plaque rupture and clinical events^6,7^. It’s widely recognized that vascular inflammation is closely linked to neovascularization, which are two interplaying key factors determining the progression of carotid atherosclerotic plaque^8^. It used to be thought that intraplaque inflammations is a “inside-out” process featured by a cascade of inflammatory responses of monocytes to the accumulation of oxidized lipid in the intima of arteries^9^. However, increasing evidences point to the process of adventitial vasa vasorum, which in turn contributes to the intraplaque neovascularization, an important feature in plaque development and vulnerability triggered by inflammation and hemorrhage^10,11^. Several serum inflammatory markers have been proposed as the risk factors for assessing the patients bearing atherosclerotic lesions of the carotid artery^2^. White blood cells constitute the effector arm of the immune system, playing the key roles in both immune surveillance and prompt response to tissue damage^2^. Studies demonstrated that different cells were found to be altered in patients with atherosclerosis, among which mononuclear cells, including both lymphocytes and monocytes subpopulations have been most frequently implicated in the pathogenesis of atherosclerosis^2^. Population-based studies have proven the association between the presence of plaque and total white blood cells and monocytes counts^12,13^.

Contrast material–enhanced (CE) ultrasound US is the useful imaging modality for visualizing the neovascularization of carotid artery plaque, due to the fact that microbubbles function as intravascular tracers allowing the identification of carotid artery plaque neovessels by dynamically assessing plaque uptake of microbubbles in the plaque^7^. CEUS can evaluate the vulnerability of plaques by quantitatively analyzing the intraplaque neo-angiogenesis and serve as a visualization diagnostic tool for the adventitia vasa vasorum^14^. However, few studies have investigated the relationship between the neo-angiogenesis and the circulating leukocytes in acute ischemic stroke patient. The aim of this study was to investigate the plaque vulnerability by quantitatively evaluating the carotid intraplaque neo-angiogenesis using CEUS in AIS patients and analyze the correlation between the cell count of leukocyte subpopulations.

## Results

### Clinical and demographic Characteristics

The demographic information, vascular risk factors and cell count of circulating leukocytes of all participants were shown Table 1. There is no significantly statistical difference between the two groups in general demographics (*P* > 0.05). The total cell count of circulating leukocytes and neutrophils in the AIS group were higher than those in the control group, while the number of lymphocytes was smaller than the control group (*P* < 0.05). There is no significantly statistical difference with respect to the level of FPG, TC, TG, LDL-C and systolic and diastolic blood pressure between the two groups (*P* > 0.05).

**Table 1:** Baseline and Clinical Data of Patients with and without AIS.

Variable	Total (n = 116)	AIS (n = 62)	No AIS (n = 54)	*P* value
Gender (F/M)	26/90	14/48	12/42	1.000
Age, mean (SD), y	66.3 (7.8)	67.7 (8.8)	64.7 (6.8)	0.121
Height, mean (SD), cm	166.7 (6.5)	166.9 (6.4)	166.6 (6.6)	0.840
Weight, mean (SD), kg	64.3 (10.9)	64.0 (11.1)	64.7 (10.6)	0.805
Body mass index, mean (SD), kg/m^2^	23.0 (3.0)	22.8 (3.1)	23.2 (2.8)	0.497
Baseline SBP, mean (SD), mm Hg	136.5 (16.3)	137.5 (15.2)	135.3 (17.6)	0.622
Baseline DBP, mean (SD), mm Hg	85.8 (10.0)	85.8 (9.3)	85.7 (10.9)	0.968
History of diabetes mellitus, n (%)	42.0 (36.2)	24.0 (38.7)	18.0 (33.3)	0.786
History of hypertension, n (%)	50.0 (43.1)	28.0 (45.2)	22.0 (40.7)	0.795
Fasting plasma glucose, mean (SD), m mol / L	5.9 (1.5)	6.1 (1.6)	5.7 (1.4)	0.407
Total cholesterol, mean (SD), m mol / L	4.6 (1.1)	4.6 (1.2)	4.5 (0.9)	0.736
LDL cholesterol, mean (SD), m mol / L	2.9 (1.0)	2.9 (1.1)	3.0 (0.8)	0.693
Triglycerides, mean (SD), m mol / L	1.5 (1.1)	1.7 (1.3)	1.3 (0.8)	0.295
Leukocytes, mean (SD), ×10^9^/L	6.57 (2.09)	7.05 (2.33)	6.01 (1.82)	0.028
Lymphocytes, mean (SD), ×10^9^/L	1.82 (0.71)	1.67 (0.54)	1.99 (0.91)	0.047
Neutrophils, mean (SD), ×109/L	4.18 (1.66)	4.59 (1.72)	3.71 (1.59)	0.018
Monocytes, mean (SD), ×10^9^/L	0.43 (0.20)	0.46 (0.22)	0.41 (0.18)	0.886
Eosinophils, mean (SD), ×10^9^/L	0.27 (0.61)	0.27 (0.58)	0.27 (0.65)	0.954
Basophils, mean (SD), ×10^9^/L	0.07 (0.25)	0.07 (0.27)	0.07 (0.22)	0.988

F indicates female; M, male; AIS, acute ischemic stroke; 1 mm Hg = 0.133 kPa.

### Quantitative Imaging Characteristics of Carotid Plaques Measured Based on Contrast-Enhanced Ultrasound Images

We examined 8 carotid artery segments (common carotid artery, carotid bulb, internal carotid artery, and external carotid artery bilaterally) in each patient, for a total of 928 segments. Conventional ultrasound and CEUS images identified at least one carotid plaques for all subjects. The conventional ultrasound examination identified 203 carotid plaques (87.5%) with 29 plaques not detected (12.5%). All the subjects had advantage plaques, which were eligible for the assessment of intraplaque neovascularization (IPN). CEUS detected the 116 advantage plaques. There was statistically significant in the amount of INP between patients with and without AIS (all *P* < 0.01) (Table 2). Semi-quantification visual assessment of the plaques found no IPN in 25 of advantage plaques (46%), and IPN in 29 plaques (54%) in control group, and in the AIS group no IPN was found in 8 of plaques (13%), and IPN was found in 54 plaques (87%). The comparison of the CEUS qualitative parameters between AIS and control subjects was shown in Table 2. The values of TIC-P, TIC-M, FC-P, FC-S and FC-AUC in the AIS group were significantly higher, compared with control group (all *P* < 0.05).

**Table 2:** Imaging Data in Patients with and without AIS.

Variable	AIS (n = 62)	No AIS (n = 54)	*P* value	*P* after adjusting for		
				BMI	SBP	DBP
TIC-P, mean (SD), dB	55.08 (14.57)	42.92 (14.63)	<0.001	<0.001	<0.001	<0.001
TIC-M, mean (SD), dB	25.29 (8.89)	21.88 (8.15)	0.046	0.050	0.042	0.044
FC-P, mean (SD)	25.24 (8.92)	23.89 (8.09)	0.041	0.051	0.053	0.047
FC-S, mean (SD), 1/s	0.71 (0.27)	0.20 (0.11)	<0.001	<0.001	<0.001	<0.001
FC-AUC, mean (SD), 1/s	17.22 (8.38)	4.40 (1.97)	<0.001	<0.001	<0.001	<0.001
**Degree of plaque INP, n (%)**						
Grade 1	8 (13)	25 (46)	<0.001	/	/	/
Grade 2	54 (87)	29 (54)	<0.001	/	/	/

TIC-P: the peak of time-intensity curve; TIC-M: the mean of time-intensity curve; FC: fitting curves of time-intensity; P: peak; AUC: area under the curve; BMI, body mass index; SBP, systolic blood pressure; DBP, diastolic blood pressure.

### Correlations between the quantitative CEUS variable and circulating leukocyte counts

**Figure 1:**
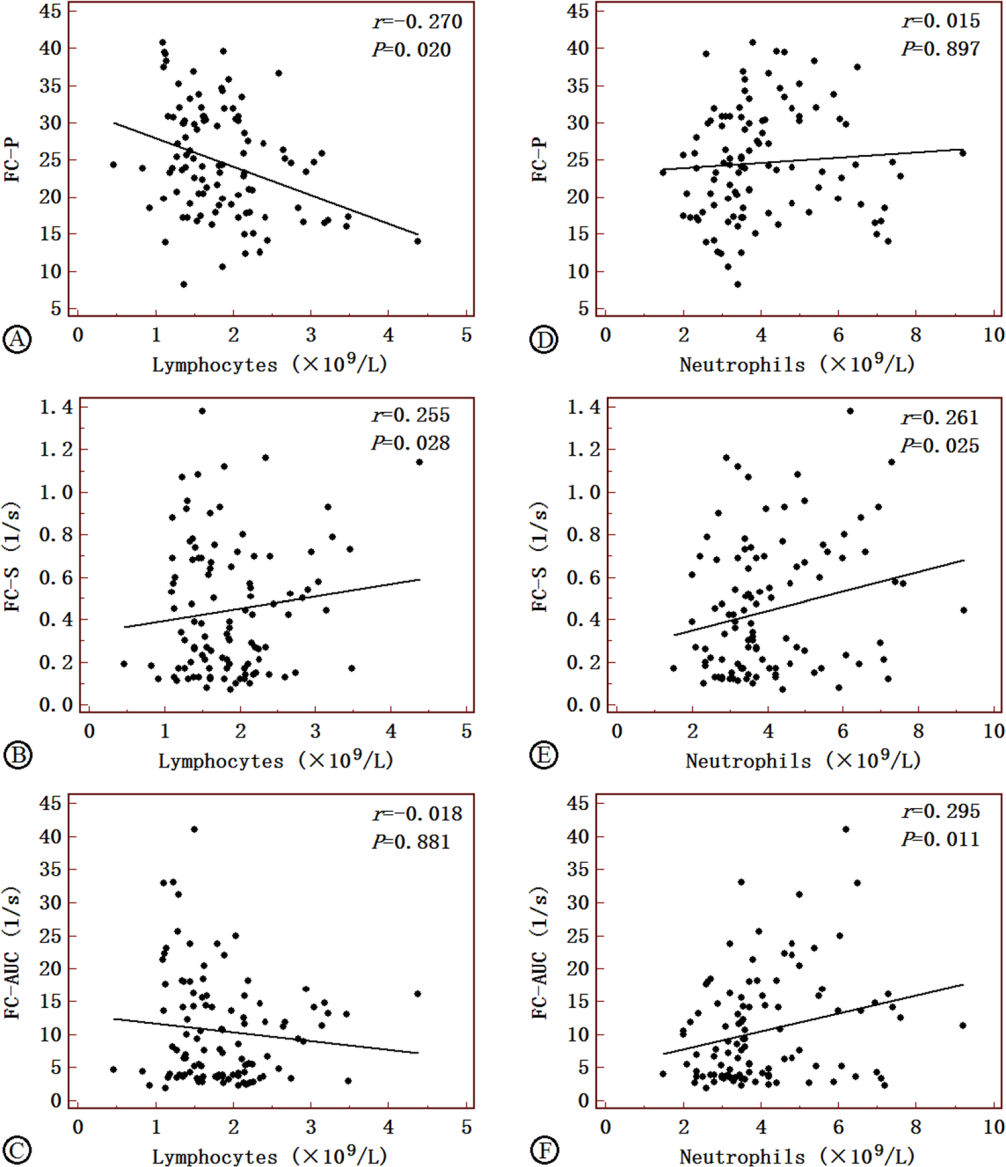
Correlations of FC-P, FC-S and FC-AUC with lymphocytes in AIS patients (**A**–**C**) and non-AIS controls (**D**–**F**). FC-P, peak of the fitting curve; FC-S, sharpness of the fitting curve; FC-AUC, area under the fitting curve; AIS, acute ischemic stroke. Correlation coefficients and *P*-value are given in the graphs.

**Figure 2:**
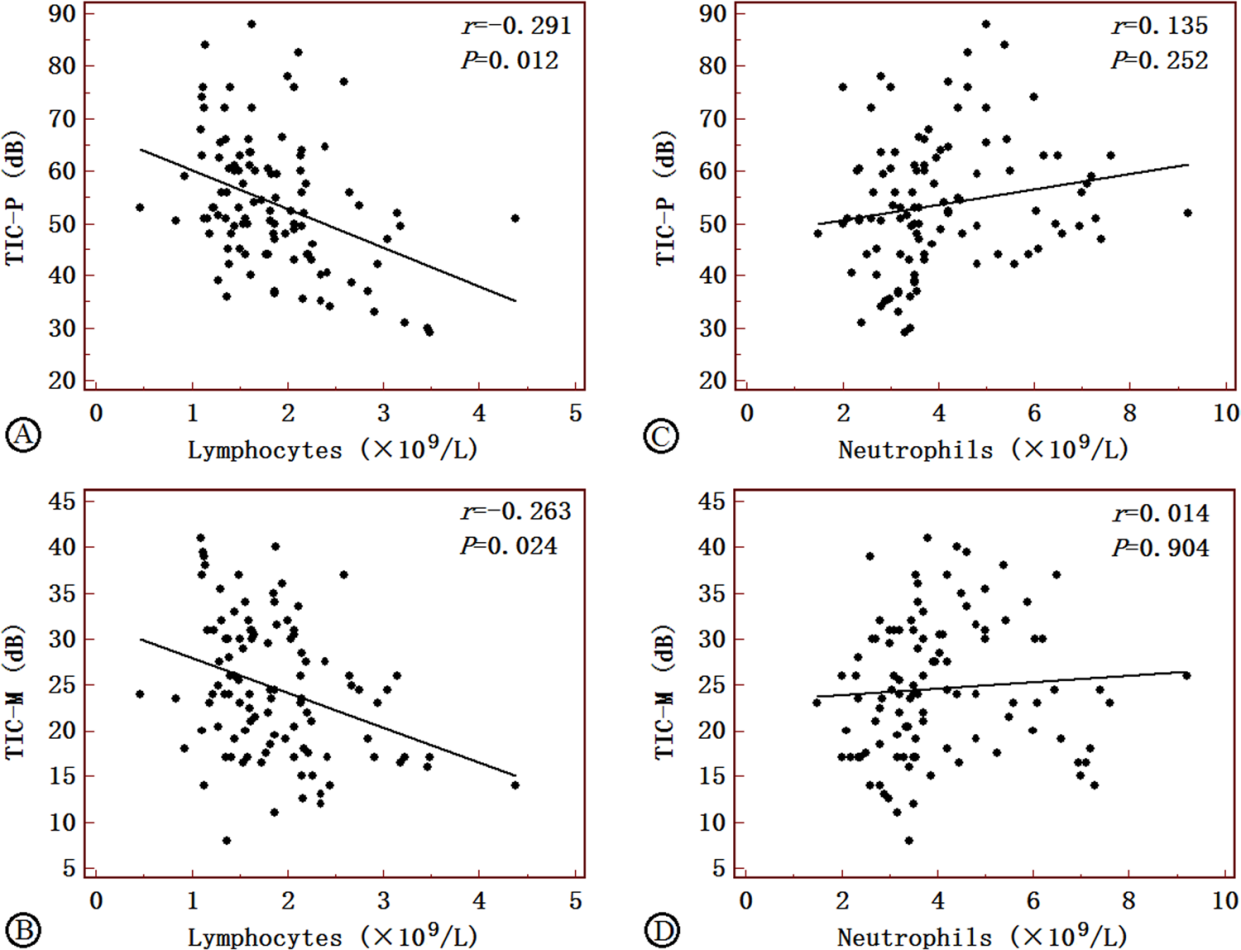
Correlations of TIC-P and TIC-M with lymphocytes in AIS patients (**A**–**B**) and non-AIS controls (**C**–**D**). TIC-P, peak of the time-intensity curve; TIC-M, mean of the time-intensity curve; FC-AUC, area under the fitting curve; AIS, acute ischemic stroke. Correlation coefficients and P-value are given in the graphs.

**Figure 3:**
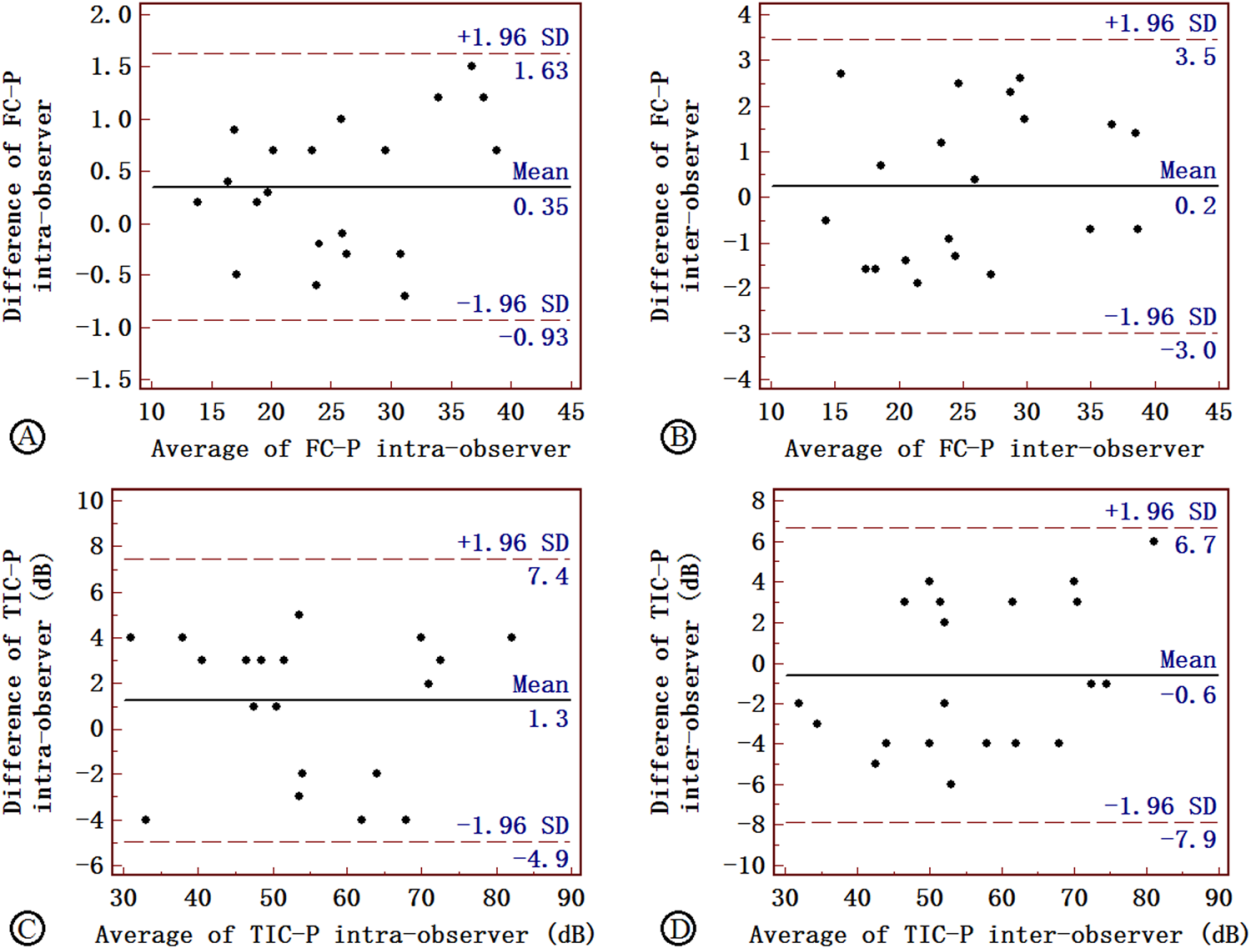
Intra- and inter-observer variability of FC-P (**A**,**B**) and TIC-P (**C**,**D**) measurements performed in 20 subjects: Bland-Altman plots showed good agreement between measurement for FC-P and TIC-P, both by the same observer (**A**,**C**) and by two independent observers (**B**,**D**).

**Figure 4:**
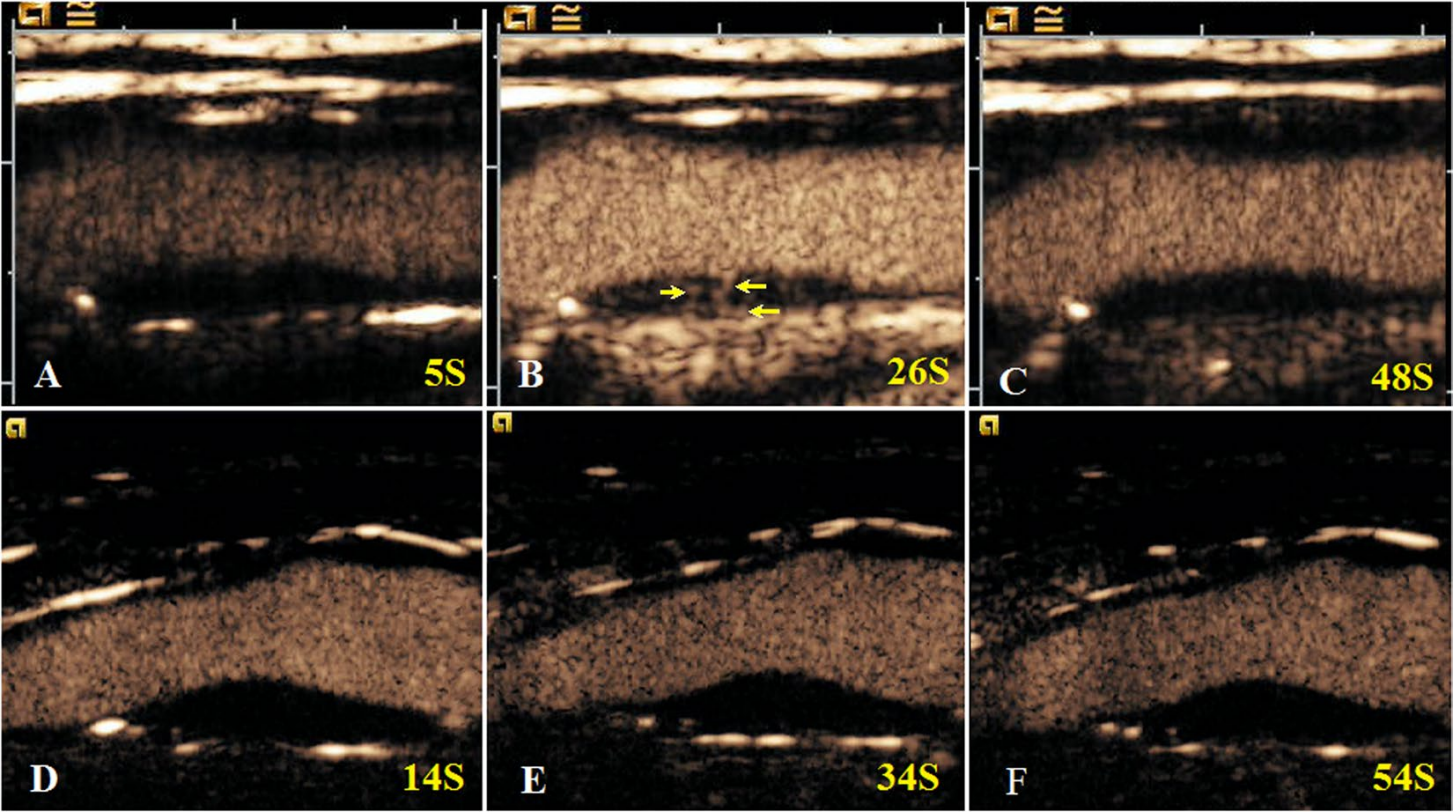
Contrast-enhanced ultrasound (CEUS) of intraplaque neovascularization (IPN) in carotid arteries of a patient with both acute ischemic stroke (**A**–**C**) and a patient without acute ischemic stroke (**D**–**F**). IPNs were observed in the plaque ROI (yellow arrows).

There was a correlation between neovascularization and lymphocytes and neutrophils, respectively (*r* = −0.223 and 0.203, *P* < 0.05). Figures 3 and 4, panels summarize the significant association between TIC-P, FC-P, and TIC-M and the average cell count of lymphocytes (r = −0.291, −0.270, and −0.263, *P* < 0.05). FC-P was negatively correlated with the level of FBG, TC, and LDL-C (*r* = −0.463 and −0.449, all *P* < 0.05) and TIC-M was negatively correlated with the average cell count of LDL-C (*r* = −0.454, all *P* < 0.05). FC-S and FC-AUC were positively correlated with neutrophils (*r* = 0.261 and 0.295, all *P* < 0.05) (Figs 1 and 2)

### Intra- and inter-observer variability

There was a favorable agreement between quantitative variables measured by the same observer and by the two independent observers for FC-P and TIC-P. The mean (±SD) difference was 0.350 (±0.653) for repeated measurements of FC-P taken by the same observer and 0.240 (±1.642) for those taken by two independent observers. The mean (±SD) difference was 1.250 (±3.160) for repeated measurements of TIC-P taken by the same observer and −0.600 (±3.705) for those taken by two independent observers (Fig. 3).

## Discussion

Cerebrovascular disease is the leading cause of deaths worldwide^15^. The rupture of carotid plaque is induced by the loss of fibrous cap integrity and luminal blood communication with the thrombogenic core of the plaque^16^, leading to the formation of a thrombus, which can subsequently embolize and occlude a distal cerebral artery, resulting in stroke^16,17^. Inflammatory response has been recognized as the most important contributor to the development of atherosclerosis and the interplay of neovascularization and inflammation are considered the main causes of atherosclerotic plaque vulnerability and rupture^5^. Plaque inflammation may have multiple effects that weaken plaque structural integrity, including inhibition of collagen production and dissolution of the fibrous matrix by means of matrix metalloproteinases^17^. In our study, we found the increasing cell count of circulating blood leukocyte and neutrophils, and the decreased number of lymphocytes in the stroke patients. The increased number of neutrophils had a significantly negative correlation with such parameters as TIC-P, TIC-M and FC-P, the quantitative imaging features, which reflect the degree of intraplaque neovascularization. There is a significantly positive correlation between the decreased number of lymphocyte and the parameters of FC-S and FC-AUC that are the imaging characteristics reflecting the permeability of the premature neovasculatures in plaques. The results indicate that some relationship may exist between the subpopulation change of circulating leukocytes and the formation of neovascularization in plaques. The cell count of a specific subpopulation of circulating white blood cells may well serve as biomarkers to predict the stability of a carotid plaque.

White blood cells constitute the effector arm of the immune system, attending to both immune surveillance and prompt response to tissue damage. It is well known that sterile inflammation is a crucial event in the pathological process that underlies atherosclerosis^18^. The formation of plaques is initiated as the deposition of oxidized lipid begins in the intima of artery wall. The enlarging plaque disturbs the arterial laminar state of blood flow and causes local turbulence or eddy in the plaque-formed vessel. The leukocytes in axial flow continuously pass through the weakened endothelium and accumulate in the lipid core to scavenge the toxic chemicals, such as the oxidized lipid. This complex cascade of pathologic processes taking place in the arterial intima ultimately lead to the ruptures of vulnerable plaques and result in brain stroke. Brain stroke can lead to prolonged inflammatory response. The leukocytes, monocytes and cytokines are recruited and react on the cranial nerve, exacerbating the damage of brain^19^.

The angiogenesis in plaque is another important pathological feature of plaque vulnerability. The neovasculatures in the plaques are comprised of immature microvessels with high permeability. Plaque instability, which leads to plaque rupture and clinical events, may be triggered by the disruption and leakage of immature neovessels originating from adventitial vasa vasorum^20^. The neovasculature growth into the plaque and increased endothelial permeability are associated with plaque inflammation, so plaque enhancement has been recognized to be a sign of plaque inflammation. Thus, developing a noninvasive imaging method to assess plaque vulnerability on the basis of plaque vascularization is highly relevant. Our study shows that the carotid artery plaques in stroke patients had significant angiogenesis than those in the control group. The quantitative parameters derived from contrast-enhanced ultrasonography, including TIC-P, TIC-M and FC-P were significantly elevated in the ASI group than the control group. Similar finding has been reported in previous studies^21^. Ultrasound contrast agent has been widely used as the tracer of the vasa vasorum. CEUS provides direct visualization of the carotid artery plaque neovascularization. The quantitative evaluation of microbubbles perfusion in the plaques could help us assess the degree of neovascularization, the indirect reflection of the inflammation, and the stability of plaques^22^. Histopathology confirmed that the uptake of microbubbles in plaques has a direct correlation with the microvessel density in the plaque. It has been reported that the increasing vasa vasorum was related to the occurrence of cardiovascular and cerebrovascular events. The inflammatory response and the angiogenesis in plaques are intertwined to aggravate the pathogenesis of atherosclerosis. The angiogenesis from the vasa vasorum in adventitia and the vascular cavity surface provide the pathway for inflammatory cells to enter the plaques^23^. Inflammatory cells can generate matrix metalloproteinases to degrade matrix, undermine the fibrous cap, and lead to plaque vulnerable^24^. Our study showed the correlation between the cell counts of blood cells and the quantitative imaging features derived from CEUS, which provide the clue for the further researches, such as evaluating the role of contrast-enhanced ultrasonography of plaque in predicting the cerebrovascular events, the relationship between the angiogenesis in different parts of the plaques and the value of peripheral blood leukocytes as the biomarker for vulnerable plaque.

Several limitations should be considered in interpreting our results. First, relatively few cases have been reported and no comparison was performed for the subgroups with and without plaque ruptures in the AIS group. Secondly, no histologic analysis was performed for the carotid plaques because of the limited availability of the plaque tissue. And thirdly, this study did not analyze the peripheral blood leukocyte subsets to explore the impact of leukocyte subsets on plaque burden. Research has shown that, circulating HLA-DR^+^ T cell levels correlates with increased carotid intraplaque neovascularization^25^. Accordingly, Ammirati *et al*. found that neovascularized atherosclerotic lesions selectively associate with lower blood levels of CD14+ and CD14^high^CD16- monocytes independently of systemic inflammatory activity^26^. Whether the change of circulating leukocyte subsets is due to a potential redistribution of these cell types into active lesions remains to be explored.

## Conclusion

Contrast-enhanced ultrasound can assess the vulnerability of carotid plaque and its acoustic parameters are closely related to peripheral blood leukocyte. The contrast-enhanced ultrasound is valuable in reflecting the inflammatory activity in the plaques.

## Materials and Methods

### Study Subjects

This prospective observational study involved 62 consecutive inpatients (48 men and 14 women, aged 53–80 years with mean age of 67.7 ± 8.8 years) treated in department of neurology, Shanghai General Hospital, Shanghai Jiaotong University. The study protocol was approved by the ethics committee of Shanghai General Hospital (2014158), Shanghai Jiaotong University (China). All participants provided their written informed consent. The methods in this study were performed in accordance with approved guideline. No incentives, financial or other, were offered to them. Patients who had the primary cerebral AIS confirmed by head CT and MRI examination within one week were included. Exclusion criteria were as follows: having the contraindications of receiving the intravenous administration of ultrasound contrast agent, such as acute cardiac failure, unstable angina, acute endocarditis, known right-to-left shunts, allergy to microbubble contrast agents; recent history of active bleeding or a malignant tumor. Fifty-four patients with imaging-confirmed carotid atherosclerosis but without cerebral cerebrovascular events were involved as the age- and sex-matched control subjects for the study group (48 men and 14 women, aged 51–79 years with mean age of 64.7 ± 6.7 years). The control group had no history of hypertension or coronary heart disease with normal findings of physical examination, echocardiography, electrocardiography and laboratory tests of hepatic and renal functions. The enrolled subjects were required to suspend the antihypertensive medications, quit smoking, drinking, and coffee for 24 hours. All the subjects underwent a conventional carotid duplex ultrasound examination followed by the contrast-enhanced ultrasound (CEUS).

Demographic characteristics, medical and familial history of the involved subjects were documented, and physical examination was performed prior to the ultrasound examination, including the measurements of body mass index (BMI) and blood pressure. The patient hypertension was diagnosed according to 2013 Hypertension Clinical Practice Guidelines^27^. Diabetes mellitus was determined based on Standards of Medical Care in Diabetes-2017. Body height and weight were measured. Blood samples were collected in the early morning following 12-h fasting for the test of fasting plasma glucose (FPG), total cholesterol (TC), triglyceride (TG), low-density lipoprotein cholesterol (LDL-C), high-density lipoprotein cholesterol (HDL-C), peripheral blood leucocytes, lymphocytes and neutrophils.

### Ultrasound imaging of Carotid Arteries

Conventional ultrasound and CEUS were performed using a Siemens Sequoia 512 and S2000 ultrasound system (Siemens, Mountain View, CA, USA), equipped with a 9-L4 linear transducer (5–9 MHz) and the software for CEUS. Carotid artery examination was performed by a trained sonography technician using a standard scanning protocol according to the American Society of Echocardiography consensus statement^28^. Briefly, B-mode ultrasound imaging and color Doppler ultrasound were performed to examine the bilateral common carotid arteries, the extracranial segments of the internal carotid arteries, and the external carotid arteries^29^. The number, location, and distribution of every carotid artery plaque was recorded, and the degree of carotid artery stenosis was assessed. Plaques were determined as advantage plaques with the following criteria: located at the distal portion of the general carotid bifurcation or at the origin of internal carotid artery; thickness >2.0 mm and the largest one was selected from the multiple lesions; no definite calcification; with the consistence of ipsilateral cerebral infarction.

CEUS examination was performed after a bolus injection of Sono Vue (Bracco, Italy) using the amplitude modulation mode. Before injection of microbubbles, the intravenous access was flushed with 5 mL 0.9% NaCl solution. 2.5 mL contrast agent was injected via median cubital followed by a flush of 5 mL saline. Contrast administration was repeatedly injected as needed with a maximal dose of up to 10 mL. The optimized contrast mode of the ultrasound imaging acquisition includes the following parameters: a mechanical index of 0.06–0.08, 90% gain compensation and 2–3 cm focus. The 90-second standard and CECU video clips were recorded and analyzed by the primary investigator (L.Z.) who was blinded to the patient demographic information.

**Figure 5:**
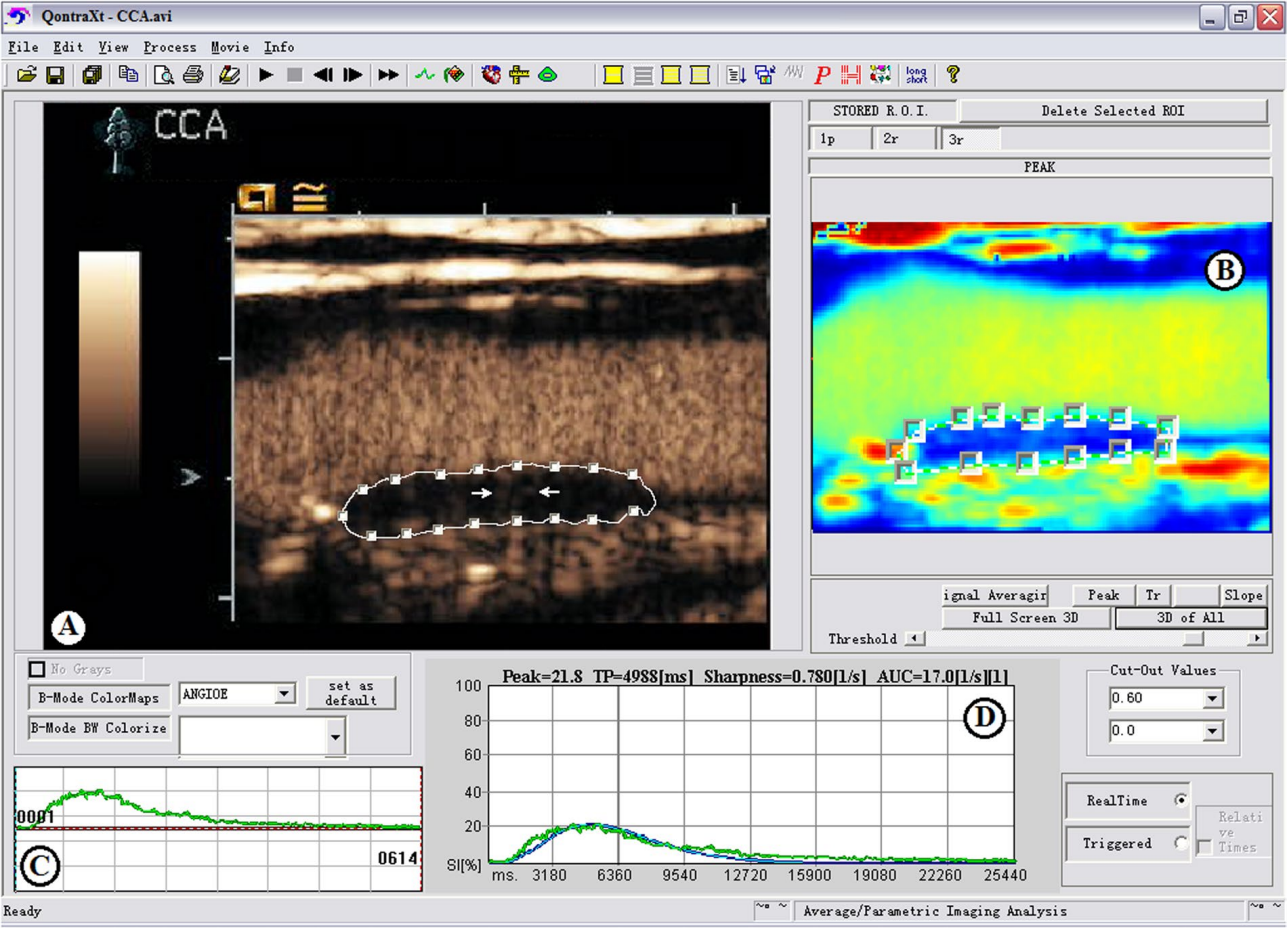
The automated quantification of intraplaqueneovasculization (IPN) of carotid plaque using QontraXt. (**A**) A manual region of interest (ROI) was placed to enclose the whole area of the plaque with IPN demonstrated in the plaque ROI (arrow). (**B**) Parametric imaging: Parametric imaging results into four images that corresponds to the maps of the curve fitting parameters. (**C**) The time-intensity curve (green-colored curve). (**D**) Parametric fitting curve: time-intensity fitting curve (blue curve). Numeric values of peak, TP, sharpness, and AUC were automatically calculated based on the time-intensity curve and are shown at the top of the graphs.

IPN was performed using a semi-quantification visual grading scale (Fig. 4). The visual grading scale was categorized as follows: grade1: no bubbles within the plaque or bubbles confined to plaque adventitial side and/or shoulder; grade 2: bubbles reaching plaque core and/or extensive contrast-agent enhancement throughout the plaque^30^. Quantification of IPN was performed using the QontraXt software (QontraXt v.3.60, AMID, Rome, Italy) (Fig. 5). The time-intensity curve (TIC) and the fitting curve (FC) for each plaque was rendered using the software. The quantitative parameters including the peak intensity (TIC-P, expressed in decibels) and the mean intensity (TIC-M, expressed in decibels) based on the TIC was measured using QontraXt software. The index peak of FC (FC-P, expressed as a percentage, maximum intensity = 100%), the sharpness of FC (FC-S, expressed in seconds^−1^) and the area under the curve of FC (FC-AUC, expressed in seconds^−1^) were used to quantitatively characterize the plaques as well^31^.

### Statistical Analysis

Shapiro normality test should be used to test normality of the distribution of variable, and continuous variables were presented as mean (standard deviation) or median [inter-quartile range]. Categorical variables are expressed as percentages. Independent *t*-test was used to compare the continuous variables between the two groups, and χ^2^-test was used to compare the categorical variables. Pearson correlation analysis was used to establish correlation between the quantitative parameters based on the measurements of CEUS images and the cell counts of leukocyte subpopulations and the correlation coefficient was rendered. Spearman’s rank correlation coefficient was used to assess statistical dependence. As body mass index (BMI) or blood pressure could have affected measurement, the results for parameters of CEUS were adjusted for these two covariates, using a general linear model. Quantitative parameters for the same plaque was measured by the same observer and by the two independent observers, and Bland-Altman analysis was performed to test the reproducibility of the repeat measurements^32^. The statistical analyses were performed using SPSS 13.0 (SPSS, Chicago, IL, USA) and *P* < 0.05 was used to indicate the statistical significance.
